# Cryptic “royal” subfamilies in honey bee (*Apis mellifera*) colonies

**DOI:** 10.1371/journal.pone.0199124

**Published:** 2018-07-11

**Authors:** James M. Withrow, David R. Tarpy

**Affiliations:** 1 Department of Entomology and Plant Pathology, North Carolina State University, Raleigh, North Carolina, United States of America; 2 Interdepartmental Program in Biology, North Carolina State University, Raleigh, North Carolina, United States of America; 3 WM Keck Center for Behavioral Biology, North Carolina State University, Raleigh, North Carolina, United States of America; University of California San Diego, UNITED STATES

## Abstract

During emergency queen rearing, worker honey bees (*Apis mellifera*) select several otherwise worker-destined larvae to instead rear as candidates to replace their dead or failing queen. This choice is crucial as the queen is the sole reproductive in the colony and her quality is essential to its success. Because honey bee queens mate with and store sperm from multiple drones, emergency queen selection presents workers with an opportunity to increase fitness by selecting full- (0.75 relatedness), rather than half- (0.25 relatedness), sisters as new queen candidates. Through patriline analysis of colonies along with large numbers of emergency queens reared by each we affirm the purported “royal” patriline theory that, instead of competing nepotistically, workers exhibit bias towards selecting individuals from particular “royal” subfamilies during emergency queen rearing events, Further, we show that these “royal” patrilines are cryptic in honey bee colonies; occurring in such low frequency in the overall colony population that they are frequently undetected in traditional tests of queen mating number and colony composition. The identification of these cryptic “royal” subfamilies reveals that honey bee queens, already considered “hyperpolyandrous,” are mating with even more males than has been previously recognized. These results alter our understanding of reproductive behavior in honey bees, raising questions about the evolutionary implications of this phenomenon.

## Introduction

The fundamental problem in the evolution of social behavior, recognized since Darwin, is that group functioning is predicated on cooperation, with individuals often incurring personal fitness costs to assist their groupmates [1]. This is perhaps best illustrated in the eusocial insects, where the vast majority of individuals within a colony often forgo individual reproduction to instead rear the offspring of one or a few queens. This life history trait represents the pinnacle of social evolution, such that a colony may be considered more as a single “superorganism” than as a collection of separate individuals [2,3]. Though defined by cooperation, the evolution of this reproductive division of labor is actually driven by multiple, and often opposing, forces of selection [4,5] and may result in conflict between worker and reproductive castes [6]. In western honey bees (*Apis mellifera*), a model system for eusocial behavior, the conflict between individual- and colony-level selection is clearly visible when the colony engages in the process of selecting a new queen [7].

As the mother of the colony, a queen’s traits have an impact on every aspect of colony functioning and her reproductive potential is crucial to colony success. If she dies or otherwise diminishes in reproductive quality, the workers must rear a replacement quickly or the entire colony will die. To do this, workers select several young (still totipotent), otherwise worker-destined larvae from the hundreds or thousands available to instead rear as queens. Workers feed these queen-selected larvae both greater quantities and a nutritionally superior diet—royal jelly—as compared to their worker-destined counterparts [8,9].

The choice over which larvae to select for emergency queen rearing is complicated by the hyperpolyandry of honey bee queens [10–12]. Honey bee queens are reported to have an average mating number of ~12 drones [13], thereby giving rise to colonies consisting of many patrilineal subfamilies. The selection of larvae during emergency queen rearing pits the self-interest of individual worker subfamilies (to have a full “super-sister” with 0.75 relatedness as the next queen) against that of the overall colony (to have the highest quality queen, regardless of subfamily; [14]). This conflict presents a rare opportunity in honey bees, where nepotistic inclusive-fitness for workers could overcome the group-level selection that has given rise to most eusocial traits.

Research to date suggests that this is not the case, however [7,15,16]. Instead, it appears that workers preferentially select larvae from particular “royal” subfamilies that are rare in the overall worker population ([17–22]; Table 1). All of these studies include one or more empirical shortcomings—such as small number of colonies, small sample sizes per colony, or too few molecular markers to fully differentiate subfamilies—each of which can make it difficult to completely determine what pattern is present in emergency queen rearing and what factors might be responsible. We addressed these logistical difficulties to determine the degree to which “royal” subfamilies are cryptic within colonies, such that they are only observed in queen rather than worker offspring. The discovery of this substantially under-recognized population within honey bee colonies has significant implications on caste determination, the collective decision-making process of reproduction, and the mating system of honey bees.

**Table 1:** Previous studies on emergency queen selection.

Study	Colonies	Samples (W)	Samples (Q)	Subfamilies	Markers
Tilley and Oldroyd (1997) [17]	3*	949	112	31 (8–15)	3
Osborne and Oldroyd (1999) [18]	4^a^	3491	802	(13–20)	2
Châline et al. (2003) [19]	2*	348	100	20 (7–13)	3
Moritz et al. (2005)^b^ [20]	8*	482	176	258 (10–59)	8
Tarpy et al. (2016) [21]	5*	376	33	102 (15–29)	8
Lattorff and Moritz (2016) [22]	3*	144	33	74.5 (10–33.2)^c^ [23]	5

Previous studies on emergency queen selection using microsatellite markers. Number of colonies sampled, Number of workers (W) and emergency queens (Q) used in each, the total number of subfamilies identified across all colonies (range for individual colonies), Number of microsatellite markers used.

^a^This study used 4 replicates with multiple (unrelated) queen-rearing units each

^b^This study presented data from colonies of both *Apis mellifera carnica* and *Apis mellifera capensis* pooled together such that it is impossible to determine from the paper how much if and to what degree the results might be driven by thelytoky in *A*. *m*. *capensis*

^c^Actual subfamilies not listed; values given are “corrected” as per Cornuet and Aries (1980)

## Methods

### Research colonies

An initial 10 colonies of *Apis mellifera* were purchased from a local commercial beekeeper on 27 March 2015 and installed at a North Carolina State University apiary in Raleigh, NC. Each queen was marked and the colonies were allowed to grow from 5 to 20 combs. During this build-up period, some colonies were removed from the study because of disease, poor condition, or queen loss as a result of unexpected swarming or supersedure. To offset these losses, an additional 4 colonies of equivalent strength were added to the experiment from previously overwintered colonies.

### Field experiment

Beginning 5 May 2015, each colony was split into one 10-frame queenright unit and two 5-frame queenless units each with eggs and young (0–2 day old) larvae suitable for emergency queen rearing divided between the queenless splits. To prevent adult foragers drifting back to the queenright units, all queenless splits were relocated to an apiary site in Cary, NC owned by the North Carolina Department of Agriculture & Consumer Services. The queenright units of each experimental unit were subsequently moved ~5 days after the splits were made (and hence after sufficient time for foragers to acclimate to their new hives) to the same apiary to facilitate the experimental procedure. Worker samples for patriline analysis were collected by placing emerging frames of brood from each colony overnight into individual cages within an incubator set at broodnest conditions (35°C and ~50% RH), sampling the newly emerged workers directly into 50 ml falcon tubes of 91% isopropyl alcohol, and stored at -20°C for further processing.

Approximately 7 days after splitting, the developing queen larvae and pupae were collected from emergency queen cells in each queenless unit and stored in individual 1.7 ml tubes at -20°C. Immediately following the collection of emergency queens, an additional frame or two (as available) of replacement larvae, including the attending nurse bees, was added to each queenless unit from its original colony source. The corresponding number of frames were simultaneously rotated back to the queenright unit (after brushing off all bees), taking care to avoid rotating brood frames that could possibly contain an overlooked emergency queen cell that could potentially emerge in the queenright unit. This process of queen sampling and frame rotation was repeated weekly until colonies failed or emergency queen rearing dwindled to <3 queens produced per unit per round. Supplemental feeding of sugar syrup was provided to the queenless units to promote maximum emergency queen rearing, and routine checks were performed to confirm that the marked mother queen remained present in their respective queenright units throughout the sampling. A total of 6 colonies produced sufficient data to analyze, yielding a total of 649 emergency queens sampled over a 10–14 week period. As the behavior under study (worker choice in queen selection) is inferred rather than directly observed, the potential for observer bias in this experiment is completely absent.

### Patriline analysis

For all samples, DNA extractions were performed in 0.2 ml strip tubes, using a solution of 150 μl 5% Chelex^®^ 100 (BioRad) in dH_2_O, plus 5 μl 10mg/ml Proteinase K. Workers were sampled by removing one metathoracic leg and chopping it up with microscissors. Queen larvae/pupae samples were made by slicing a small piece off each frozen larva/pupa using razor blades that were flame-sterilized between samples. Reactions were run in a thermocycler under the following conditions: 60 min at 55°C, 15 min at 99°C, 1 min at 37°C, 15 min at 99°C, then held at 4°C.

For genotyping, multiplex PCRs were performed to amplify 8 microsatellite markers (A24, A76, A88, A113, Ap43, Ap81, ApJC2, and B124) [24–26] using 1 μl of supernatant DNA template, 5 μl multiplex PCR Master Mix (Kapa), 0.67 μl combined fluorescent-tagged primers, and 3.33 μl lab grade H_2_O, for a total reaction volume of 10 μl. Reactions were run in a thermocycler under the following program: 3 min at 95°C, followed by 48 cycles of 15 s at 95°C, 30 s at 57°C, 45 s at 72°C, with an elongation step of 5 min at 72°C and held at 4°C. PCR products were then diluted 10:1 with lab grade H_2_O. For fragment analysis, 1 μl of dilute PCR products was combined with a 9 μl solution of Gene Scan Liz 500 sizing standard (Thermo Fisher) in Hi-Di Formamide (50 μl Liz 500 per 900 μl Formamide), denatured for 5 min at 95°C, and sequenced on a 3730 DNA Analyzer (Applied Biosystems) by the NCSU Genomic Sciences Laboratory (Raleigh, NC).

Microsatellite markers were analyzed using GeneMapper^®^ v4 software (Applied Biosystems), with scoring assignments verified through visual inspection. For each colony, the queen genotype was deduced from, and patrilines inferred for, the individual samples as per Mendelian segregation. Individuals matching both heterozygous queen alleles at a given marker, such that it was impossible to assign a given allele to the queen or the drone, were assigned parsimoniously to whichever possible subfamily had the highest number of other members to avoid disproportionately inflating the numbers of uncommon subfamilies or create “phantom” new ones in the data [27]. Similarly, individuals with missing data at a given marker were grouped into the largest possibly subfamily assignment for all other loci. Because of variability in DNA extraction and PCR amplification, analysis ranged from 4–7 usable microsatellites per colony.

## Results

**Fig 1:**
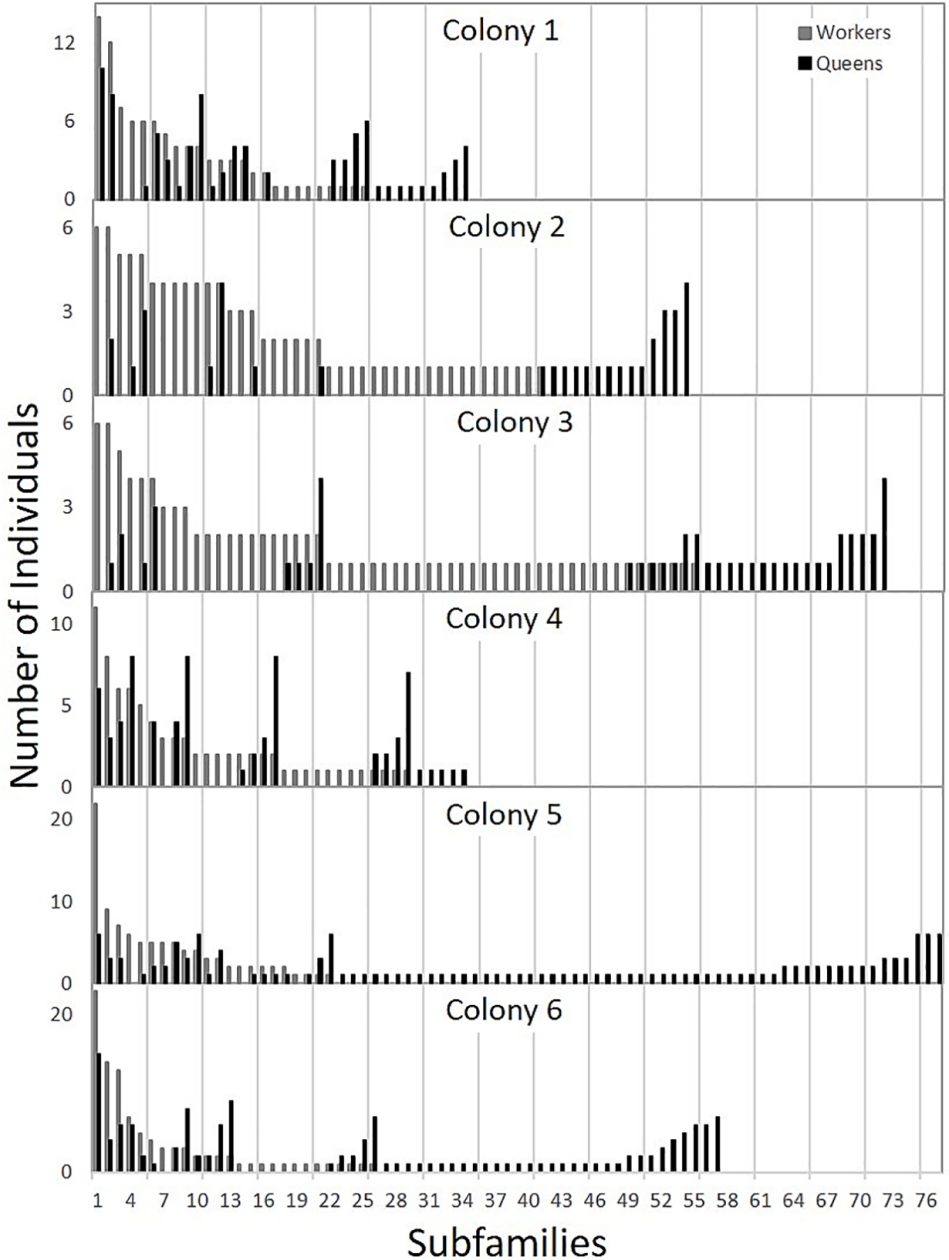
Subfamily distribution per colony. Subfamily distribution of workers and emergency-reared queens in six honey bee colonies. *Grey bars* are the counts of workers in each colony. *Black bars* are the counts of emergency queens from the same subfamilies. Subfamily counts per colony range from 34–77, with many queens deriving from patrilines rare or absent in workers sampled.

Genotype analysis of the 6 experimental colonies identified 327 total subfamilies (34–77 per colony) from a total of 552 workers and 512 queens. This included 108 subfamilies (4–40 per colony) exclusively detected in workers and 130 subfamilies (5–55) exclusively detected in queens. An average of 40.21% of the queens produced per colony were from queen exclusive “cryptic” subfamilies (Fig 1, Table 2).

**Table 2:** Summary of colony data.

Colony		Workers	Queens	Subfamilies	W_Ex_	Q_Ex_	Percent Q_Ex_	Me (T)	Me (W)	Me (Q)	Markers
**1**		93	85	34	8	9	17.65%	18.89	16.09	20.29	4
**2**		96	35	53	33	13	60.00%	42.79	38.65	24.83	6
**3**		96	47	72	40	17	51.06%	64.68	57.01	47.04	6
**4**		77	70	34	14	5	7.14%	19.41	19.91	15.99	5
**5**		94	135	77	4	55	62.96	32.43	11.88	65.08	6
**6**		96	140	57	9	31	42.45%	19.32	9.64	29.15	7
**Average**		**92**	**85.33**	**54.50**	**18.00**	**21.67**	**40.21%**	**32.92**	**25.53**	**33.73**	

Colony number; Number of workers and queens genotyped; Subfamilies detected per colony; Subfamilies exclusively detected in workers (W_Ex_) and queens (Q_Ex_); Percentage of queens from Q_Ex_ Subfamilies; Effective queen mating number (as per [13]) calculated from total samples (Me (T)), workers only (Me (W)), and queens only (Me (Q)); Number of microsatellite markers used in analysis

**Fig 2:**
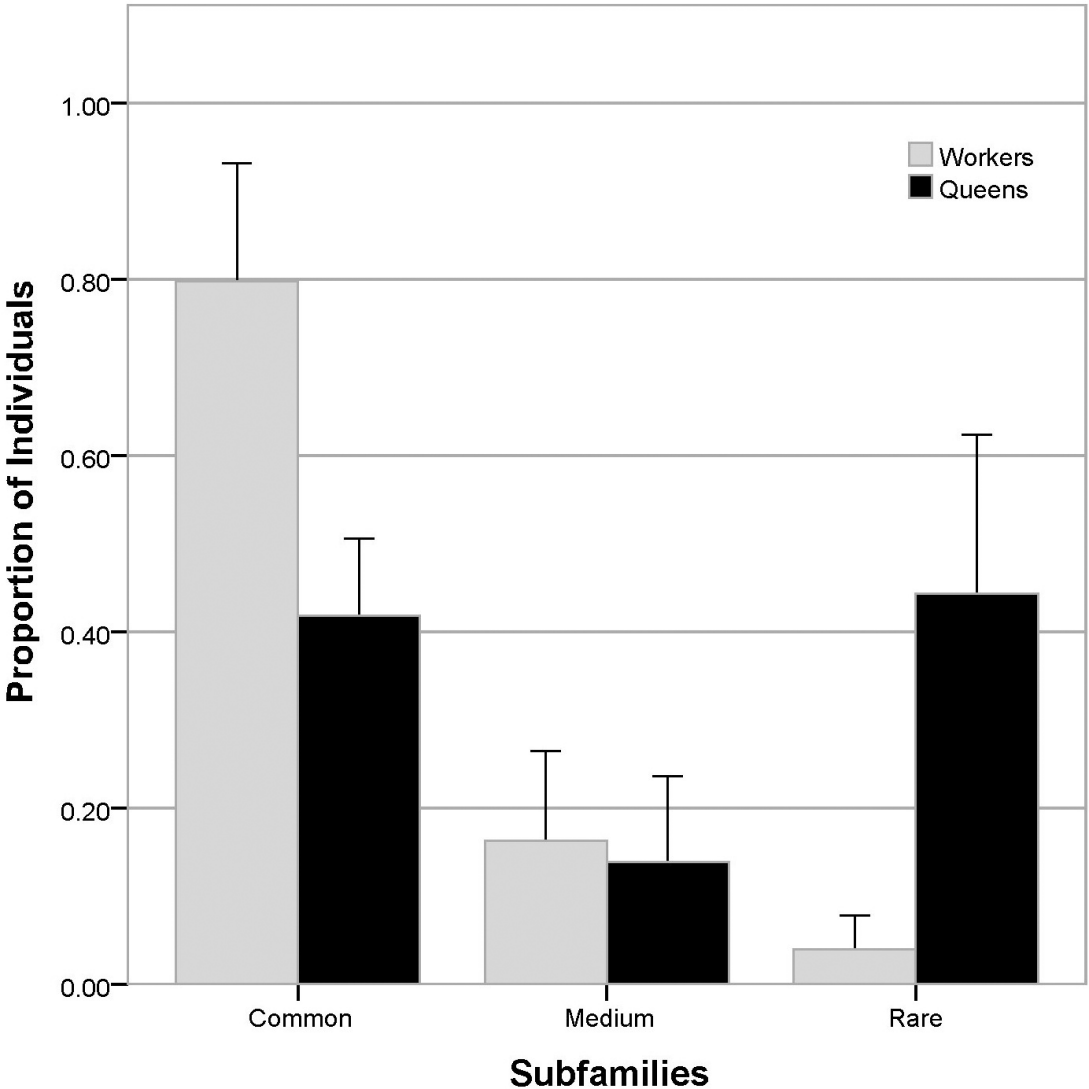
Frequency distribution of subfamilies. Tertile distribution of subfamilies, grouped by worker abundance. Subfamilies from each colony separated into three equal groups (common, medium, and rare) based on worker (grey) abundance, allowing for comparison across colonies with unequal numbers of subfamilies. Emergency queens (black) paired with corresponding worker subfamilies. Observed worker and emergency queen populations are significantly different (p<0.0001, Fisher’s exact test).

For statistical analysis across colonies, the subfamilies in each colony were grouped into three equal tertiles—common, medium, and rare [22], with the most abundant third (ranked by their proportional representation in the worker populations) labeled “common,” intermediate third labeled “medium,” and least abundant third labeled “rare” (Fig 2). Statistical analysis confirmed that there is a significant difference between the subfamily distributions of workers and emergency-reared queens (p<0.0001, Fisher’s exact test). An additional analysis using the same groupings but restricted to patrilines represented by three or more members confirmed that the significance of these results is not driven by the high number of patrilines with only one or two members detected (p<0.0001, Fisher’s exact test).

## Discussion

The results of this experiment provide the most thorough evidence to date documenting the nature of “royal” subfamilies in honey bees, demonstrating that they are both pervasive across colonies and cryptic to the point of remaining undetected with typical sampling and analysis. If these results accurately reflect the subfamilial makeup in colonies of *Apis mellifera*, they cast into doubt the established concepts of honey bee reproductive behavior such as the degree of polyandry in honey bee queens. Possible alternate explanations include experimental flaws in the sampling of workers or queens, the presence of more than one queen in an experimental unit, errors in analyzing the molecular markers, or some sort of unknown biological effect that would give rise to this sort of pattern. None of these conditions seems likely, however. No additional samples of emerging workers were taken throughout the field experiment, as subfamilial distributions in honey bee worker populations remain stable over short periods of time [10,17,28] without any indication of sperm clumping or cryptic female choice [29]. Polygyny with the presence of a daughter queen in addition to the mother could possibly explain some of the data, and temporary polygyny of mother and daughter queens is sometimes observed in honey bees [30,31], but we can rule that out as a sufficient explanation based on genetic and circumstantial evidence. The original marked queens were consistently identified throughout the time each colony was inspected and sampled for queen larvae, no unmarked queens or queen cells were identified in the queenright units despite frequent observation, and all of the colonies were severely weakened or completely dead by the time observation ceased, making it extremely unlikely that extraneous queens were present and undetected. Even if an additional laying queen was present for a short period of time and went undetected visually, the clear pattern of “royal” patrilines across all 6 colonies indicates that it is not merely an artifact of cryptic polygyny as these subfamilies are not consistent with Mendelian granddaughters. As for alternative biological explanations, thelytoky bears consideration but, while well documented in the Cape Honey bee (*Apis mellifera capensis*) subspecies [32–34] it is not present (or exceedingly rare) in European stocks. Other speculative possibilities, such as induced changes in the appearance of microsatellites or some sort of non-Mendelian marker segregation are unsupported by the relevant research to date.

Given that the validity of these findings appears to be robust, there are two immediate implications that revise our understanding of honey bee reproduction: (1) there is a cryptic population of queen-biased patrilines within colonies that are consistently underrepresented in molecular-based estimates of honey bee polyandry, and (2) honey bee queens are mating more than previously recognized, as traditional mating estimates are calculated from worker populations using biased samples that often exclude the rare “royal” patrilines. If actual queen mating numbers are as high as we have detected here (Table 2; Fig 1), then honey bee queens would be better described by “extreme hyperpolyandry” than merely hyperpolyandry. At this point it remains unclear whether extremely high mating numbers in honey bee queens have simply been overlooked or whether this phenomenon could be a response to environmental and management conditions, as increased genetic diversity promotes colony functioning and ability to overcome disease and other stressors [33–39]. While the extremely high mating numbers detected here are remarkable for *Apis mellifera*, previous mating estimates for western honey bees have been relatively low for the genus *Apis* in general [13] and the numbers we have observed are on par with reported estimates for *Apis nigrocincta* [40] and lower than those for *Apis dorsata* [41].

The results presented here clearly indicate a non-nepotistic pattern of larval selection among nurse honey bees during emergency queen rearing (reviewed in [7,42]), but the underlying mechanism of the selection process of larvae for raising queens remains largely unknown. Factors of larval attractiveness involved in the worker choice do include pheromonal components, as workers exhibit a pheromonally mediated preference for well-fed larvae during emergency queen selection [43] and brood are also known to pheromonally regulate worker development and behavior [44]. Two such compounds, methyl-oleate and ethyl-oleate, are produced in much higher amounts by queen larvae as compared to worker larvae [45], and methyl-oleate in particular bears further scrutiny as it is also a component of the worker-attractant retinue pheromone produced by adult queens [46]. Emergency queen selection may be viewed as larval competition as much as worker selection, as any advantage in attracting “royal treatment” from nurse bees during the critical hours of larval selection would confer a huge potential fitness boost on those larvae [22,47]. Such an advantageous trait would not be unprecedented, as similar larval competition has been documented in other eusocial hymenoptera such as *Acromyrmex* and *Prystomyrmex* ants [48,49].

While this “royal” patriline effect plays a major role in emergency queen rearing, this is only one of three conditions under which honey bees rear new queens. The primary condition under which new queens are reared is when the colony reproduces through swarming [50]. During these events, the mother queen lays eggs in special “queen cup” cells and the resulting larvae are invariably raised as queens. Shortly before the virgin queens emerge, the mother queen and approximately three-quarters of the workers leave the hive and establish a new home elsewhere [51]. Because swarm queens are raised directly from queen-laid eggs, there is little opportunity for workers to bias the outcome. Such activities would be limited to preferentially transferring eggs/larvae from worker cells into queen cups [52], or selectively rejecting certain queen-deposited eggs/larvae cannibalism or destruction of completed swarm cells prior to queen emergence [21]. Lattorff and Moritz [22] found that swarm queens do not significantly differ from worker patriline distributions, though their data suggest that further analysis may be necessary to rule this out entirely. Similarly, supersedure queens (produced when a queen is failing but still present and laying) are presumably also laid by queens in queen cups [53] and so should not undergo significant selection by workers. Because of this, the “royal” patriline bias is a primary factor in only a minority of requeening events.

The relative rarity of queens produced from direct worker selection in this way may be a reason why these “royal” patrilines remain rare. Any heritable trait that increases the odds of becoming a queen would quickly sweep to fixation and thus cease to be rare in a population unless there is a significant tradeoff involved [18]. One factor countering this affect is the lack (or at least significant reduction) of worker-induced patriline bias during swarming and supersedure queen rearing. The actual mechanisms driving the preference for “royal” larvae in emergency queen rearing have yet to be investigated so we cannot rule out potential mechanistic explanations such as polygenic or epigenetic effects, though a mechanistic understanding of this preference would not directly address the apparent selection counteracting this preference and maintaining these patrilines as “rare” in honey bee populations. Such selective explanations might include some sort of reproductive tradeoff between males and females, such that the advantage in queen rearing is counterbalanced by a reduction in drone fitness due to linked or single-gene effects such that the “royal” drones are generally outcompeted by their typical rivals. Alternately, there could be a tradeoff between reproductive (individual queen) traits and worker (colony) traits. Honey bee subfamilies exhibit variation in different task specializations and proclivities, providing beneficial diversity to colony functioning [35,36,54,55], and subfamilial variation in reproductive traits has been identified before although not in the context of queen rearing [56]. It is possible that the same evolutionary forces that gave rise to caste segregation are at work, maintaining rare “royal” families that produce high-quality queens but low-quality workers at low abundance in the overall population.

A complex behavior such as queen selection—with its huge implications on future colony survival, relative subfamily fitness, and individual fitness of the candidate larvae—is certainly evolving under the pressures of multiple forces of selection. While many of the specific details and mechanisms are still to be determined, at this point we may safely conclude that, while inclusive fitness for nepotism may favor the individual level during emergency queen rearing, that advantage is profoundly overridden by opposing selective forces acting at multiple levels favoring cooperation and altruism. While selfish actions can still exist in the most highly social organisms [57], the evolutionary road to eusociality necessarily suppresses individual interests to the advantage of the group.

## Supporting information

S1 File Sample genotypes. (XLSX)
